# The work and relatedness of ties mediated online in supporting long‐term condition self‐management

**DOI:** 10.1111/1467-9566.13042

**Published:** 2019-11-25

**Authors:** Chris Allen, Ivaylo Vassilev, Anne Kennedy, Anne Rogers

**Affiliations:** ^1^ NIHR Collaboration for Leadership in Applied Health Research (CLAHRC) Wessex School of Health Sciences University of Southampton Southampton UK

**Keywords:** social networks, online communities, self management, long‐term conditions, digital health, qualitative methods

## Abstract

The ‘care transition’ is characterised by reduced state involvement in chronic illness management in response to socio‐political movements aimed at meeting the challenges presented by an increased prevalence of chronic illness. Amongst these changes has been online communities’ rising importance in everyday interactions and attention is being increasingly paid towards the ways online contacts might contribute to self‐management. Whilst research has illuminated the relevance of personal networks in long‐term condition management, it is relevant to extend this work to consider the place of ties mediated online in this bricolage of support, including better understanding the work drawn from them and the strategies involved in eliciting it. This study examined the work and relatedness of 30 participants, who used online communities. Participants were asked about the role of on and offline ties and ego network mapping was used to frame conversations about the nature of this support. The context of engagement followed three main themes. Participants drew from online communities in response to deficits in offline support, they used online ties to leverage support or action from offline ties and they used online ties to substitute offline support, with less intimate online ties.

## Introduction

How care is thought about, organised and delivered has, over the last two decades changed dramatically. This has been largely driven by increased longevity, a shift from an acute to chronic disease profile and attendant social and economic pressures. In a process described as a ‘care transition’ (Bury and Taylor 2008), new forms of emergent care include an increasing role for patients as self‐managers, with a shift in the locus of responsibility for health from the state to the individual with the condition. The latter has required further revision to the critiques made by medical sociologists, about the traditional conceptualisation of the sick role (Parsons 1951) and its relevance to how people respond to conditions which cannot be cured, and which must be managed over time (Crossley 1998, Gerhardt 1989, Varul 2010). Research has pointed to how those with a long‐term condition (LTC) more typically engage with self‐management activities in the context of managing in the here and now, which includes a need to maintain a sense of equilibrium, personal ability and responsibility in order to fulfil their obligations to normative social roles, whilst also minimising the extent to which life becomes too orientated around illness (Brooks *et al*. 2015, Entwistle *et al*. 2018, Greenhalgh *et al*. 2011).

Despite the evidence for the normalising approaches individuals take to self‐management in their everyday lives, official approaches often focus on addressing peoples’ purported knowledge and motivational deficits (Gately *et al*. 2007, Hibbard and Gilburt 2014, Protheroe *et al*. 2008), whilst paying little attention to what people strive for in order to live well, or how this can be achieved in messy everyday life (Entwistle *et al*. 2018, Morgan *et al*. 2017). Peoples’ own self‐management strategies extend beyond narrow, professionally advocated forms of management, which in primarily focussing on disease control being achieved through adherence to tightly defined ways of managing, often poorly align to what people themselves see as important (Entwistle *et al*. 2018, Morgan *et al*. 2017). People's own preferential strategies are influenced by the value ascribed to certain activities, social and contextual factors and the inclusion of wider personal network involvement and access to relevant network resources (Greenhalgh *et al*. 2011, Rogers *et al*. 2014, Vassilev *et al*. 2017). Indeed, whilst policy has increasingly promoted the importance of individual responsibility, the foundational work of Christakis and Fowler (2007) amongst others, disrupted the assumption that individual behaviours are the cornerstone of health practices. This has furthered research examining the relevance of people's social networks to condition management (Perry and Perscosolido 2012, Rogers *et al*. 2014, Vassilev *et al*. 2013). Access to online support, through online communities,^1^ is increasingly seen as part of this bricolage (Allen *et al*. 2016, 2019) and may provide a means through which further distance is created from the assumptions inherent in the sick role, both in terms of the person assuming the sick role (their rights and obligations) and the waning of medicines previously dominant position, through increased lay access to relevant information and advice (Hardy 1999). Despite the relevance of both on and offline ties in shaping self‐management practices and access to related work and resources, to date these have been poorly considered together. A relational understanding of online ties in self‐management requires knowledge of the full diversity of the on and offline ties that contribute and the situations in which they are called on, which is the focus of this paper.

Prior research has shown that in the context of LTCs, successful management requires the availability of, and ability to locate and negotiate relevant network resources (Vassilev *et al*. 2014). Often this work has concentrated on the role of kin, which is consistent with research demonstrating the centrality of intimate family processes (e.g. Gallant *et al*. 2007, Graven and Grant 2014). Whilst evidently important, they only reveal a partial view and account of the full range of linkages relevant to self‐management practices (Rogers *et al*. 2014). With the recognition that the work involved in keeping someone well is becoming increasingly specialised and complex, there is a need to understand wider network involvement, including the role of a wide range of network members in response to the diversity of problems that those managing a LTC must overcome.

Examining how people decide who to turn to implicates a process that is purposeful (Kennedy *et al*. 2015, Perry 2012). Needs are ascertained and decisions are made about who is best placed to meet them (Perry and Perscosolido 2012, Vassilev *et al*. 2014). This is both contextual and adaptive, resulting in practical and purposeful intent towards the activation of specific ties to meet certain needs. Prior research has pointed towards the increasing role for online ties in self‐management practices (Allen *et al*. 2016, Ziebland and Wyke 2012) catalysed by the social reach the internet affords and the ease within which new connections can be made (Ellison *et al*. 2014). This has resulted in the likelihood of individuals with the capabilities to draw on them in meaningful ways, having greater choice and control over who contributes to their management; through the emergence of opportunities to bypass offline ties that are either not available, difficult to access or unwanted.

In the face of the range and diversity of actors and processes involved in an increasingly complex division of labour, there is a need to better understand our more networked life and the negotiations around health in this context. There is currently a deficit in understanding as to the role that online ties serve within personal networks as a whole. With ever increasing access to new forms of collective support online, it is important to understand the context and circumstances in which online and offline ties are called upon, and the network strategies that those living with a LTC adopt when able to access a wider pool of potential on and offline contributors. In this regard, this research responds to calls to better consider the means of support that people have available to them and their contribution to self‐management (Entwistle *et al*. 2018).

## The study

To aid the systematic reporting of our findings, the consolidated criteria for reporting qualitative studies guidelines (Tong *et al*. 2007) was followed.

### Aims

This study aimed to describe the nature of engagement with online ties among those who use them to support LTC self‐management, as well as identify how people saw the contribution of online ties, in the context of their personal network as a whole.

### Sample

Purposeful sampling was used to recruit 30 participants living in the Wessex area of the UK, who used online communities to support the self‐management of a physical LTC. Several approaches were used to identify and recruit suitable participants, including seeking permission to actively promote the study through condition specific online communities and local groups. Those who expressed an interest in participating in the study were screened against the inclusion/exclusion criteria (over 18, living in the Wessex area of the UK, able to speak English and give valid consent, living with a LTC and using the internet to support self‐management) and provided they consented, were recruited into the study.

Participants had a range of LTCs, representing diverse condition experiences including Type 1 Diabetes, Parkinson's disease, Polymyalgia Rheumatica (PMR), Giant Cell Arteritis, Fibromyalgia, heart problems, Multiple Sclerosis (MS), Interstitial Lung Disease (ILD), Myalgic Encephalomyelitis (ME), chronic pain, Lupus, HIV, Stroke, Hepatitis C, Asthma, Stiff Persons Syndrome (SPS) and Epilepsy. It was common for participants to have more than one condition, including those relating to mental health.

Whilst the original focus of recruitment was on those using online communities to connect with peers in condition specific groups, most participants discussed the relevance of more general social media platforms (such as Facebook and Twitter), that facilitated access to new and existing ties; both of which were seen as relevant to self‐management. All participants had a formal diagnosis, but the interviews included reflections on periods prior to this, such as the conditions initial genesis. The participant's ages ranged from 25 to 74 and the mean age was 52. Participants were predominantly on lower incomes (53% having an income lower than £26,500), but highly educated (56.7% having a degree) and most didn't work, either due to their condition (*n* = 7), or because they were retired (*n* = 13).

### Data collection

Data were collected over 18 months using semi‐structured biographical interviews (Roberts 2002) to elicit a chronological narrative of the ties activated on and offline in response to self‐management needs. This supported a more complete visualisation of the context of engagement within a wider system of support to emerge. A convoy model was used to map the participants ‘ego’ network and was used primarily as a heuristic device, to gain a better understanding of the people and/or groups considered to be important to the participant's everyday self‐management. This approach was adapted from Pahl and Spencer's (2010) work on personal networks, used in previous research examining the diversity of illness related work (Brooks *et al*. 2016, Vassilev *et al*. 2013).

Using this approach, participants placed network members in one of three circles, representing: important, more important and very important (the inner most circle). This allowed networks to be described according to the importance of members’ contributions, allowing for a detailed visualisation of different ties contributions, in a way that negated the tendency for a positivist assignment of roles, based around relationship type, whilst also acknowledging the possibility of complimentary and overlapping roles (Kennedy *et al*. 2015). For each network member placed, participants were asked about their contribution, as well as why they turned to this person or group. The interviews lasted between 45 and 180 minutes and took place in either the participant's home, at the University of Southampton or at an agreed local facility. They were audio recorded and transcribed verbatim.

### Analysis

Data analysis was carried out concurrently with data collection as part of an iterative process. As with Brooks *et al*. (2016), illness work (Rogers *et al*. 2011), was used as a conceptual starting point to the analysis, following which, inductive thematic analysis was used to identify the strategies used to draw illness work from on and offline ties. Transcripts were read multiple times to ensure familiarity with the data, prior to the initial coding, which was carried out by several authors from a range of disciplinary backgrounds including Nursing and Medical Sociology. Authors met regularly to discuss the on‐going analysis and to explore and confirm the emergent themes, before the final three themes were agreed on. At this point, it was decided, that no further recruitment was required.

### Ethics

Ethics approval was granted by the University of Southampton's Ethics Committee.

## Results

### Network placement and attributional meaning of the internet

Of the 30 participants, 24 identified an online contact or group within their personal network of support as being important to everyday management. In contrast, offline support groups were less frequently present in the participants networks (*n* = 11) and participants had limited access to people offline, with the same condition (*n* = 5). When present in the network, partners always appeared in the inner circle (*n* = 21) and contributed towards everyday work (such as domestic support) but were largely excluded from conversations directly relating to management.

Healthcare professionals were present in 28 of the participant's networks, but were only in half the participant's inner circle, suggesting a side lining of formal care, relative to other ties. The qualitative analysis further illustrated that the participants often saw their healthcare professionals as essential, but only in relation to narrowly defined roles that had to be carefully curated, for example, the provision of a prescription or their role in onwards referral to other services.

In mapping the participant's network, where possible, distinction was drawn between ties already known offline and new ties formed online. However, whilst placement within the concentric circles mostly resulted in groups and contacts being seen as either ‘online’ or ‘offline’ contacts, further exploration revealed a more complex process of suffusion. Contacts initially established online, frequently became offline contacts. Likewise, contacts already known offline, often featured in online networks too and were often called on online.

Online ties were accessed through a diverse range of online communities. Those targeted through their shared experience of the condition, were sought through groups hosted on platforms specifically related to health (such as Health Unlocked) or through condition focussed groups on more general platforms (such as Facebook and Twitter). Online access to strong and weak ties already known offline, was typically through general online communities (such as Facebook), though more intimate online media, such as WhatsApp was commonly used to coordinate intimate tie involvement during crises.

The results, which are presented below, indicated the use of online ties in network, and illness management strategies. The context of engagement followed three main themes. Participants drew from online communities in response to deficits in offline support, they used online ties to leverage support or action from offline ties and they used online ties to substitute offline support, with less intimate online ties.

### Online network extension in response to unmet offline needs

Online communities allowed participants to employ proactive strategies to shape their network with a variety of ties that they perceived to be best able to meet their needs at specific times. By using online communities, they were able to reach out and extend their network in response to perceived deficits in several lines of work which related to current, as well as forecasted needs.

Scant encounters with formal, unresponsive healthcare systems presented as a feature of online engagement and were particularly visible in the conversations around the period between the initial genesis of a condition and formal diagnosis. In conditions that were difficult to diagnose, it was common for the participants to face long periods of time in which they had no access to formal health care. Often during these times, online peers were the only support they could access:

So then I went on the forums and um, I mean one of the forums … ‘Limbo land’ … you go to if you think you might have MS but you have not been diagnosed for definite … and then like people who have had it for a long time will go onto that forum and basically help. (P15, Female, 30–40 years old, MS)

Even at the point of diagnosis, participants often received poor or incomplete explanations of their condition and how it should be managed:

I don't just mean I knew nothing about Parkinson's, I didn't know anything about medication, I didn't really know anything about symptoms, I didn't know anything about support. I knew literally nothing at all. (P12, F, 50–60, Parkinson's disease)

Incomplete explanations and use of inaccessible medical terminology, often meant participants were unsure of what exactly they were supposed to be doing. This lack of translation resulted in participants having to speak to people online to understand what was discussed, which included understanding why certain behaviours and regimes should be followed. Eventual changes in habits were typically driven by online peers who took the time to translate abstract biomedical concepts, into understandable and relevant ones:

I didn't know that I needed to check my blood sugar that many times a day. I was told I needed to check. But I didn't know why. I never thought to ask. I didn't really understand the numbers on the screen. So, I would check and see like 15 [mmols] and not really know that that was way too high. So, I started to think, you know. Why am I checking? (P3, F, 30–40, Type 1 Diabetes)

Access to a performative online stage (Bullingham and Vasconcelos 2013) in which the self‐management practices of others were made visible, provided a reference point situating many of the activities of self‐management as acceptable, normal, and something other people did. This was particularly influential following a diagnosis. Being able to speak to others about why they measured their blood sugar, helped translate what had previously been seen as a pointless activity, into an activity in which the importance was clearly understood, resulting in its adoption.

Lack of timely access to formal, specialist care was also relevant. It was common for participants to face long waits to access a specialist and during this time, online communities were often the only condition specific support they were able to access. Beyond diagnosis, more formal support was rarely available when questions arose, or specific problems were encountered. Often these problems were not seen by respondents as serious enough to seek urgent care, but nonetheless required urgent solutions, especially where problems impacted upon overriding everyday concerns:

Yeah, if I have got a problem, I will go there (online community) first. Mainly because I can only see the consultant every few week's. (P8, F, 60–70, PMR)

Participants rarely had access to offline contacts with the same condition. Thus, whilst many felt that their offline intimate network provided emotional support, it did not come from a place of shared understanding. Online ties were often seen as better placed to offer this support, through their shared understanding of the condition and its impact on daily living:

They try, but as hard as they try to understand, they won't ever understand what it's like. Like even though they are amazing at it, um … they won't truly understand. I think like, I need somebody that actually gets it … you know, even just one person. (P15, F, 30–40, MS)

Lack of network members with the same condition also limited the availability of offline experiential knowledge, which was relevant in helping participants overcome challenges relating to the condition and its management. Participants deliberately sought out people experiencing similar everyday challenges relating to specific symptoms, complications and activities such as work, sport, pregnancy etc. (Hartzler and Pratt 2011):

I haven't officially put people into [boxes], but I will know that they are like the tech groups and there are kind of eating disorder groups and exercise groups … I just put people into their little files and I file them away until I need them. (P3, F, 30–40, Type 1 Diabetes)

Such a strategy reflected the broader everyday needs of those managing a LTC, which were often at odds with the narrow set of self‐management behaviours promoted by formal care, in order to control the condition and prevent it from getting worse. The need to minimise the impact of the condition on everyday life, often resulted in the participants taking steps to ensure that they had access to people with diverse experiential knowledge to meet immediate needs. These connections were often maintained overtime through the awareness that their knowledge might become relevant in the future:

So, like for me, if I was looking to have children, again that's going to be a new thing for me, me now exercising a lot, it's a new thing for me. So, I need advice from someone who knows what it's like and what they do. (P9, F, 30–40, Type 1 Diabetes)

In the participant's narratives, there was a visible mismatch between the self‐management activities that were promoted by formal care and the steps that people took to minimise the impact of their condition on daily life (Entwistle *et al*. 2018). It was common for participants to deviate from their sick role obligations, through rationalised non‐adherence (Demain *et al*. 2015). Deviance from these obligations often reflected formal self‐management approaches being unresponsive and insensitive to what people valued and how they wanted to live (Entwistle *et al*. 2018). Generally, participants were aware of the biomedical markers that professionals used to appraise compliance with their prescriptive and narrow instructions, but they resisted these becoming their overriding concern. Instead of discounting medical advice altogether, participants adopted a modus vivandi, by using formal advice in combination with the experiential knowledge of online ties, to arrive at approaches that ‘would do’, if they allowed for more meaningful ways of living (Brooks *et al*. 2015, Entwistle *et al*. 2018):

And if you go to the Diabetes website, it advises you that when you get in from a night out, you have a bowl of pasta. I am not going to sit there at 3am in the morning making pasta. I am going to have cheesy chips or a kebab. (P3, F, 30–40, Type 1 Diabetes)

In adopting a range of other people's trial and error approaches to everyday management, participants gained a sense of control over the choices they made and were able to adapt practises in accordance with individual priorities. Contra the traditional sick role regimes that impact upon desired ways of living, rigid self‐management practices were considered in relation to their future benefits, which were by no means guaranteed. The incompatibility and overriding everyday concerns of participants made compromises necessary. These were facilitated by the activation of online ties with a set of experiences relevant to the adoption of approaches to self‐management that accommodated participation in, specific activities (e.g. going on holiday) and biographical events (new employment, child rearing etc.). This knowledge plugged the absence of lay and critical experiential expertise in participant's offline network, providing opportunities to compliment the prescriptively narrow, empirically focussed advice of formal care:

I would say that the online stuff compliments the primary care. Because I think the primary care gives me … It gives me the insulin, it gives me the basics that I need to, you know, keep me alive, but the online stuff gives me the knowledge to tweak it, to make it work best for me. (P7, F, 30–40, Type 1 Diabetes)

In being more reliant on empirical truths, the advice formal care was able to offer, was often seen as being too narrow, too limited and far too risk averse to be useful on its own. The advice online ties were able to offer, often went beyond what the participants felt professionals could feasibly provide. Often this was not medical advice per se, but relevant to decisions about everyday situations that were made challenging by illness (Hartzler and Pratt 2011), such as attending a job interview, getting the train etc. This experiential knowledge, which helped people to navigate the aspects of daily life made difficult by illness, was often unavailable in the participant's personal networks, creating a deficit that was necessarily addressed through online engagement with those who had successfully found a balance. Since the participants felt that this could only come from someone with these experiences, online ties facilitated an adaptive approach to everyday self‐management that would have been difficult to achieve without being able to locate and reach out to new online ties to mitigate deficits in access to this type of knowledge offline.

### Online community engagement as leverage of offline tie action

Online communities were used to leverage support from existing offline professional and lay ties in situations where the participants felt they were being unresponsive to their needs.

Activity with online ties was used as leverage for formal services, for example in prescribing medications and/or referrals to specialist care, and access to others online improved individual's capacity in dealing with these situations. Conversations with a pool of people online, led to a wider understanding of possible treatment options, which helped participants negotiate treatment that best reflected their needs. These conversations helped frame needs with a view towards securing certain resources. Thus, online communities were used as a backstage (allowing for knowledge acquisition and rehearsals) to help prepare the way individuals demonstrated candidacy for certain treatments (such as a new medication or device), new equipment (such as perching stools) and financial support:

And then that made me think: ‘ah, ok, I need to be proactive and I need to push my GP for other tests and other possibilities. (P15, F, 30–40, MS)

Seeing what others with similar needs were offered (such as specific interventions, equipment, certain medications and new technology such as insulin pumps), helped participants negotiate candidacy towards resources that they felt would be beneficial.

Conversations online also helped leverage support from lay offline ties. Many were frustrated with their family and friends not understanding their condition, how it is managed or its impact on daily life. Whilst frustrations were partly addressed by talking to other people online who ‘got it’, online conversations were also useful in leveraging offline support, particularly in providing illness reification, which involved strategies that deliberately front staged their condition, including making visible conversations with online peers with the same condition. Presenting themselves in this way, particularly for those with invisible or contested illness, had relevance in legitimising their condition and its associated difficulties, in the face of both lay and medical scepticism (Barker 2008).

Impression management saw participants revise or adjust their online presentation of self, to offline ties, with a view of signalling current and future needs (Bullingham and Vasconcelos 2013). This was done for example through the sharing of videos, pictures or articles (identity indicators) about the condition (Bullingham and Vasconcelos 2013); or through posting pictures and videos of themselves receiving treatment to areas where it was likely (and hoped) that offline ties would see, such as Facebook. This reinforced support needs and thus, leveraged support:

They forget … the only time they ever really remember is when I put the pictures of me receiving the [Intravenous Immunoglobulin] (IVIG), because I do that. Because I think, actually ‘hello’, I am actually ill, I am poorly and then all of a sudden they remember … all of a sudden people will be like ‘is there anything we can do?’, ‘do you want us to pop over … social media has helped me remind people. (P30, F, 40–50, SPS).

Such actions were made relevant by people offline poorly understanding their condition. Because network members were often unable to differentiate between acute and chronic illness, participants were concerned that reduced involvement in other aspects of life, might be seen negatively by offline ties. This was common during periods of intense treatment, or when the condition was making participation in other aspects of life difficult and necessitated the need to reassert legitimacy, recalibrate the expectations of others and address waning interest and support.

Participants used sites such as Facebook to talk about their condition for the first time with less intimate offline ties, allowing them to express parts of themselves that they had previously suppressed. The greater levels of editorial control offered by online platforms allowed for a degree of impression management not available offline (Bullingham and Vasconcelos 2013), which supported disclosures around illness that would have previously been difficult to openly discuss (Suler 2016). In this context, online platforms operated as a ‘safe space’ in which participants could talk about their condition and its impact on their life. Some of the participants likened this to ‘coming out’, or in the context of Parkinson's ‘dropping the P bomb’:

I was gradually starting to just talk a little more about, you know, my health, or not being able to do something and feeling frustrated or being so tired or whatever, but never actually, you know, going I have this and this is my situation. Um … and then I got my wheelchair and I was like, right, people are going to see me in my wheelchair, right, I am going to post a photo of myself in my wheelchair, on Facebook. (P2, M, 30–40, ME)

These actions acted to buffer against future emotion‐focussed work. Attempts were made to educate others, in the hope that through ‘preventative telling’, future disparaging attitudes and difficult conversations might be averted (Link *et al*. 1989, Scrambler 2009). Indeed, education is seen as a strategy for coping with felt stigma and a way of managing other's reactions to labelling (Link *et al*. 1989, Scrambler 2009). Offline, such disclosures clearly bring about risks of direct discrimination, but in providing a safe space, online communities helped people get a sense of how others may react to their condition, at a distance, and in a way that was less threatening and painful than telling people individually, with the asynchronous nature of online communication and the lack of visual cues, helping to limit their exposure to people's reactions (Suler 2016). In addition, by making offline ties aware of their condition, the participants hoped that future support could be leveraged using these sites.

Indeed, being able to access offline ties through online platforms, supported the leverage of tangible support, such as lifts to hospital; emergency childcare etc. This was done in a way that matched needs with availability and a desire to help:

So, I did have an emergency appointment come through a while ago and couldn't get a baby sitter, so I just said on Facebook, you know, ‘can any of my friends have the boys for just an hour’ and my friends around the corner said, ‘oh no worries, I am free’ … there is a lot of interlocking support. It makes coordinating things easier. (P29, F, 30–40, Fibromyalgia)

Status updates provided a means through which needs could be signalled to a broad audience, allowing for requests to be targeted at the participants entire local network, which meant the participant did not feel like a burden, because support was both matched with availability and volunteered from a diverse range of supporters.

### Substitution of offline emotional work with online ties through identity management and protective avoidance

The availability and ease of accessing online ties, led to offline ties being substituted for those online, to support identity management and reduce the burden placed on others. This was common when participants required either minimal involvement from offline ties (i.e. when minimal needs were comfortably met online), or when participants feared overburdening friends and family. Most participants did not want to be defined by their illness and labelling was a common concern, especially when it was felt that it might negatively influence the way others saw them; placing at risk their existing role and identity within their network (Charmaz 1983, Link *et al*. 1989). The availability of online ties provided opportunities to negotiate support away from people that they were close too, who participants felt themselves responsible for. This helped participants to maintain a sense of normalcy and control over relationships:

I tend to keep the Diabetes thing away; I tend to keep it to the Twitter account, but my Facebook, that's my close friends and family … I don't really want them to have as much access to it, I don't want them to have as big an insight into this, but the online community, I don't really mind. (P9, F, 30–40, type 1 diabetes)

Perceived threats to normative social roles and the way in which existing ties evaluated them, saw individuals suppressing external markers of illness and withdrawing from intimate support; which helped to maintain their positive self‐evaluations; whilst also limiting the extent to which others (particularly intimate ties) could devalue them (Scrambler 2009). Felt stigma, which emanated from the fear of being discredited in the eyes of others, saw a reduced role for intimate ties traditionally implicated in support, who were substituted for readily available online ties, whose availability and ease of access provided the opportunity for aspects of condition related support to be negotiated at a distance.

However, with online communities constituting a normative way for keeping in touch with friends and family, participants were compelled to present different versions of themselves, to different online audiences with selective disclosure of self, reflecting participants’ use of impression management (Bullingham and Vasconcelos 2013). Multiple accounts were used to keep condition specific needs at a safe distance. Condition specific needs were often met through using a pseudonym, but when one was not used, participants often deliberately posted to areas online where they felt friends and family were unlikely to see their comments. In adopting multiple presentations of self, through an awareness of different audiences operating in different contexts (Bullingham and Vasconcelos 2013), participants were able to maintain face and promote a consistently positive image of themselves, by presenting the negative aspects of their condition at a distance, reducing the perceived threat of devaluation from people they knew offline (Scrambler 2009). Traditionally the employment of secrecy and withdrawal, whilst warding off many of the negative aspects of labelling, reduced access to network support and resources. Indeed, the fear of people reacting negatively towards the person with a LTC, has been shown to reduce social interaction and help seeking behaviours (e.g. Shechtman *et al*. 2016), leading to smaller, more concentrated networks that are less responsive to people's needs. In this context, online spaces provided a new way for individuals to shape the manner in which they presented themselves to others, allowing for greater control over how different ties came to see them, based on the context of their relationship.

Whilst substitution was used to maintain the essence of existing relationships, many of the participants found moral value in being able to cope with certain aspects of their condition without implicating their friends and family (Brooks *et al*. 2015). It was common for participants to consider who they would and would not turn to. Prior research has shown a reluctance to seek help from offline ties (Allen *et al*. 2016, Sanders *et al*. 2011) and in this case, offline contacts (particularly adult children) were actively avoided, through fears of overburdening them, and often in recognition of their own pressures in life. At the same time, protective avoidant strategies were employed to safeguard the valuable contribution of those providing support offline, who for most participants due their proximity, provided most of the everyday illness related work, especially domestic work. These protective network strategies safeguarded the availability of this essential work, that some participants relied on:

Because they are so vital, I don't want to be the one that moans and groans and I am not saying people don't have that right, because of course they do, but I would rather not have that element with them, because we are so close … it's not that I am hiding anything, it's just, if I am going to have a moan about something, I will do it on there. (P13, F, 50–60, ILD)

You can speak to people who you don't know, so you don't really care so much. I know that sounds awful, but to some extent they are expendable. (P29, F, 30–40, Fibromyalgia)

In being aware of the strain the condition placed on existing relationships, participants instead drew on willing and available online ties, who provided opportunities to move aspects of support that did not require proximity, away from the offline ties that the participants were concerned of overburdening; reducing the risk of these relationship becoming too strained, and breaking down. Access to people online and the ease of which support could be negotiated, resulted in support often being sought there, especially since the contribution of online ties was accompanied by fewer obstacles associated with offline support (Sanders *et al*. 2011). In contrast to offline support, the overconsumption of online support was not an active concern, due to the number of potentially supportive ties that were available.

You are worried about burdening your friends and family. I previously would have sat and like thought very hard about whether I should phone someone and who I should phone, like who did I call last time? (P29, F, 30–40, fibromyalgia)

Dependence on friends and family was generally seen negatively, especially when it was unreciprocated, and participants wanted to be seen as coping with minimal involvement from others. Thus, strategies reflected a desire to reduce the extent to which the participant became too dependent on a small number of friends and family and involved the substitution of support that did not require proximity, with similar support online. This reluctance to implicate others, is perhaps unsurprising in a society which frequently privileges individual capacity over collective effort, alongside the current focus of policy, which calls on those with a LTC to be independent, considerate, health conscious and responsible citizens (Ayo 2012, Ellis *et al*. 2017). In being distant, less tangible and in feeling at times ‘less real’, online ties fostered a sense of independence and control over condition management, which allowed for often minimal involvement from others, or the reduced involvement of those providing large degrees of support to sustainable levels.

## Discussion

These findings bring into view the elements and configuration of a new, digitally mediated stage of care transition reflecting the changing nature of support in response to the social affordances of more participatory forms of the internet. In this new modus operandi, connected individuals are situated as the central foci in their network and can use this position to meet their needs by drawing on a mixture of on and offline ties (Rainie and Wellman 2014). This is supported through increased access to a range of resources and increased opportunity for multiple presentations of self, employed as a strategy to secure network resources in ways that allow for the maintenance of existing roles and relationships (Bullingham and Vasconcelos 2013).

Under conditions of complexity, the participants used agentic strategies that were purposeful, adaptive and future looking to overcome support that was unavailable, unwanted, or that required leverage to be realised. Having access and the ability to appropriately draw on often quite informal resources, provided an enabling context for self‐management that allowed for adaptive responses to formal cares more limited offering, both in terms of access and purpose (Entwistle *et al*. 2018). Through affording those managing a LTC more choice and control in the everyday management of their condition, including the opportunity to reduce the involvement of the intimate ties traditionally implicated, this digitally mediated stage of care transition sees participants’ increasingly managing illness on their own terms. This has shifted both professional and lay roles, with work increasingly being taken up by supportive ties online.

The participants took responsibility for their own health and were knowledgeable about their condition, through accessing lay and professional sources of information, which they used to tailor their approach to self‐management around the way in which they wanted to live (Entwistle *et al*. 2018). However, whilst the participants fitted the idealised notion of a ‘good self‐manager’ (Ellis *et al*. 2017), their approaches to self‐management did not always fall in line with professional expectations, nor those placed on someone assuming the sick role (Varul 2010). Where participants did not expect a return to full health, their concerns and the way in which they managed their condition, reflected a desire to minimise the conditions disruption to their everyday lives. Prolonged conformity with the set of narrow self‐management activities promoted by professionals, placed at risk valued aspects of people's lives and the way in which they wanted other people to see them. This led to participants seeking out alternative sources of lay and experiential information, where strictly following medical advice was neither feasible nor appropriate. The participants were often able to confidently leverage required actions from healthcare professionals, through the knowledge they acquired from online peers in supporting the acquisition of resources that might help them achieve more acceptable ways of managing.

Research has alluded to the empowering processes of online communities (Brady *et al*. 2017) and earlier work in this area has pointed towards the increased access to health information that the internet affords, as being likely to bring about such changes in the role and place of formal care in LTC management (Hardy 1999). Patient choice is no longer based solely on the options presented by formal care; with an increasing role being seen for online ties in this context. This is relevant, as formal self‐management support has often been criticised as not accommodating the ‘messiness’ of people's lives (Ellis *et al*. 2017) or acknowledging the importance people place on other aspects of their life, which may be threatened by narrow approaches to self‐management (Entwistle *et al*. 2018). The range and diversity of support people can now call on and the varying ways in which they can do it, has addressed some of these tensions; offering those living with a LTC a degree of flexibility that has typically been absent in prescriptively narrow approaches to self‐management (Morgan *et al*. 2017).

New online ties had a greater or lesser salience in different contexts and existing ties could be leveraged to provide support in new ways. On and offline networks often operated as overlapping fields with their own internal logics. Online ties were often deliberately kept at a distance that was ‘just right’ (Miller 2016). Their fleetingness, ease of negotiation and gifting nature, meant that this support was often seen as always being available, irrespective of the level of effort directed at supporting these relationships. This contrasted offline ties which required purposeful effort to maintain but were often essential. As a result, online involvement could be ‘powered up’ and ‘powered down’ (Perry 2012), creating flexible personal networks capable of providing a buffer to the fluctuating needs of those managing chronic illness. This was articulated in the frustrations the participants had in trying to access formal care in times of need, positing online networks as an adaptive response to deficits in formal care. As such, online ties, due to their condition related expertise, were often called on to provide information that once would have been largely the province of now more marginally involved healthcare professionals.

Offline ties often lacked the knowledge and experience to respond in the same way as online peers and thus were rarely turned to for advice. However, they necessarily provided support that required physical, as opposed to digital proximity, such as helping with cleaning, shopping, transportation etc. when the participants were unable to. This work was essential and the awareness of supportive ties online provided a means to which the saliency of this work could be maintained. This relational work has been seen previously in the context of weak ties, through which involvement is seen to limit the extent to which relationships are adjusted to accommodate illness and in turn supporting a less condition related focus to important relationships (Rogers *et al*. 2014). Thus, there was a narrowing of the role of intimate ties in support, to that which could not be accommodated by online ties.

### Implications and future directions

Deficits in contemporary healthcare articulated in the participant's struggles with accessing formal care and dissatisfaction with the narrow approaches to self‐management may be met through the conversion of a range of different contributions from on and offline ties, into meaningful capabilities that help people live well with a LTC. These findings illuminate a hidden and under‐explored patient system of implementation that has supported people in the adoption of more holistic approaches to self‐management. In illuminating the participants ability to draw on a range of resources to develop approaches to self‐management that worked well for them, this paper highlights the need for the professional support in self‐management to be moved away from existing approaches that see people as lacking in the necessary knowledge, skills and motivation to adequately manage their condition, towards instead considering peoples capabilities (such as digital skills) and how they can be enhanced in order to support approaches to management that are acceptable.

Future work might consider how to best understand and make use of the hidden work that people themselves do to manage their condition, but this will require the removal of the negative and moral judgements that are placed on people who deviate from professional expectations around self‐management (Hill 2010). Doing so, may allow for movement away from prescriptively narrow views of self‐management support, towards collaborative approaches that focus on the person living with a LTC and their everyday priorities (Entwistle *et al*. 2018).

The focus of recent policy suggests an accelerated push towards self‐management activities being realised through engagement with online resources (Hunt *et al*. 2015). Whilst the possible benefits of these resources are becoming more visible, many (including online communities) require reasonably sophisticated digital skills in order to be used meaningfully. It would be naive to assume universal access to this way of managing, because access and ability are so unequally distributed (McAuley 2014) and people's ability to manage in this way, will be affected by many of the same social constraints affecting other self‐management practices (Greenhalgh *et al*. 2011, Vassilev *et al*. 2017). Those lacking either access or the ability to draw on online resources in this way, may lack the necessary network infrastructure to be able to adapt to this refashioned patient role and in being more reliant on support being available through offline networks, are likely to be further marginalised if issues of access and use are not addressed.

### Limitations

The responses were drawn from a mixed group of participants, who lived in the South of the UK. These participants were mostly well‐educated and as a result, often presented as digitally able. This was unintentional and reflected the characteristics of those who consented.

Research would benefit from better understanding the sociocultural factors that underpin these practices, particularly in marginalised groups, who would benefit from being the focus of future research in this area.

The inclusion of previous stages of illness, reflected the importance of considering what Morris and Sanders (2018) describe as ‘critical moments’ that call for the mobilisation of different network resources. Whilst the interviews focussed on stages of management and featured discussions about online ties at key moments (such as the initial genesis, diagnosis, crises, flare up etc.), interpretive caution is required when considering these past events, which are of course subject to issues of recall.

## Conclusion

Demographic and epidemiological transitions have created a situation in western countries, including the UK, whereby socio‐political movements have reduced state involvement in LTC management, whilst increasing the focus on patients self‐managing their own condition. To date what has largely been promoted is a set of narrow behaviours and expectations around how LTCs should be managed, that poorly align to people's own expectations around health and how they want to live. There has emerged an increasing role for the internet in supporting self‐management practices, particularly in plugging offline support found wanting, either through not being available, or not considering the broader everyday needs of those managing a LTC.

To our knowledge, this is the first study to look at online health related work in the context of ones total configuration of condition related personal network support, including the context and circumstances of engagement with online ties. Unsurprisingly, offline support continues to be essential where physical presence is required, but the internet gave people the opportunity to realise support away from professional and lay support to manage their condition on their terms. A concern is that this transition places those lacking ability to draw on these resources appropriately, at a disadvantage. Understanding how those from more marginalised communities adapt to this changing landscape of care should be the focus of future research.

## Note

1 Definitions of a ‘community’ generally point towards a group of communicating individuals, who share support and have a sense of collective identity (Gzurd *et al*. 2016). Online communities represent technologically mediated opportunities for geographically disparate and proximate people with common interests and needs to form connections and share support and resources (Hammond 2016).
